# Mechanisms of cell death induced by arginase and asparaginase in precursor B-cell lymphoblasts

**DOI:** 10.1007/s10495-018-1506-3

**Published:** 2018-12-21

**Authors:** Lucy E. Métayer, Richard D. Brown, Saskia Carlebur, G. A. Amos Burke, Guy C. Brown

**Affiliations:** 1 Department of Biochemistry, University of Cambridge, Tennis Court Road, Cambridge, CB2 1QW UK; 2 Department of Paediatrics, University of Cambridge, Cambridge, CB2 OQQ UK

**Keywords:** Arginase, Asparaginase, Leukaemia, Apoptosis, Cell death

## Abstract

Arginase has therapeutic potential as a cytotoxic agent in some cancers, but this is unclear for precursor B acute lymphoblastic leukaemia (pre-B ALL), the commonest form of childhood leukaemia. We compared arginase cytotoxicity with asparaginase, currently used in pre-B ALL treatment, and characterised the forms of cell death induced in a pre-B ALL cell line 697. Arginase and asparaginase both efficiently killed 697 cells and mature B lymphoma cell line Ramos, but neither enzyme killed normal lymphocytes. Arginase depleted cellular arginine, and arginase-treated media induced cell death, blocked by addition of arginine or arginine-precursor citrulline. Asparaginase depleted both asparagine and glutamine, and asparaginase-treated media induced cell death, blocked by asparagine, but not glutamine. Both enzymes induced caspase cleavage and activation, chromatin condensation and phosphatidylserine exposure, indicating apoptosis. Both arginase- and asparaginase-induced death were blocked by caspase inhibitors, but with different sensitivities. BCL-2 overexpression inhibited arginase- and asparaginase-induced cell death, but did not prevent arginase-induced cytostasis, indicating a different mechanism of growth arrest. An autophagy inhibitor, chloroquine, had no effect on the cell death induced by arginase, but doubled the cell death induced by asparaginase. In conclusion, arginase causes death of lymphoblasts by arginine-depletion induced apoptosis, via mechanism distinct from asparaginase. Therapeutic implications for childhood ALL include: arginase might be used as treatment (but antagonised by dietary arginine and citrulline), chloroquine may enhance efficacy of asparaginase treatment, and partial resistance to arginase and asparaginase may develop by BCL-2 expression. Arginase or asparaginase might potentially be used to treat Burkitt lymphoma.

## Introduction

Despite the increasingly good outcome in childhood leukaemia, relapsed and refractory ALL still represent the major causes of death from childhood cancer. Since most drugs used in the treatment of ALL target DNA replication, surviving patients face a long-term legacy of complications, including a fourfold increased risk of developing a second primary malignancy [1]. Asparaginase is integral to treatment of leukaemia worldwide, demonstrating the feasibility of clinical treatments by enzymatic amino acid depletion.

The arginine dependency of tumours was recognised in the 1970s and is currently being studied in various cancers mainly using the *Mycoplasma* enzyme arginine deiminase ADI [2–6]. The clinical usefulness of arginase was felt to be limited due to its short in vivo half-life, high K_M_ and optimal pH around 9 [7, 8]. However, pegylation allows successful in vivo use, including studies with T-cell leukaemia [9, 10] and AML [11].

Arginine depletion can inhibit cell proliferation due to uncharged tRNAs activating protein kinase GCN2, or ER stress activating PERK, to phosphorylate initiation factor eIF2 [12]. eIF2 phosphorylation blocks translation of virtually all mRNAs, but potentiates translation of GCN4 and ATF-4 [13, 14]. GCN4 upregulates amino acid synthesis and protein degradation, promoting survival. However, ATF-4 translation induces CHOP expression, down-regulating anti-apoptotic Bcl-2 and up-regulating pro-apoptotic TRB3 and DR5 [15, 16]. Arginine deprivation can induce autophagy, in part via mTOR [5, 17–22] which is normally protective [5, 18, 21–23], although excessive autophagy can induce cell death.

Although there are an increasing number of studies with arginase in cancer, B lymphoblastic malignancies have not been well examined. We have previously briefly reported that arginase induced cell death in a human pre-B ALL cell line, 697, but not a human mature B ALL cell line, Tanoue [24]. However, the mechanism by which arginase induces cell death of lymphoblasts is poorly understood, having been described as necrotic [11, 25] or apoptotic [6, 9, 22, 23, 26, 27], without any evidence that blocking apoptosis prevents cell death. The role of autophagy in arginase-induced death is also unclear [23, 28]. The mechanism of cell death is important because the inflammatory and immunological consequence of cancer cells dying by apoptosis, necrosis or autophagy are very different [29], and also has implications for what other agents might potentially be used for co-treatment.

The mechanisms by which asparaginase induces cell death of lymphoblasts is also not entirely clear, despite its routine use as therapy for B ALL. In particular, there is uncertainty as to: (i) the role of autophagy, (ii) mechanisms of resistance, and (iii) the relative roles of the asparaginase and glutaminase activity of this enzyme in inducing cell death [30].

In this study, we compared the mechanism of cell death induced by arginase and asparaginase in pre-B lymphoblasts. We find that both enzymes induce cell death by apoptosis, but the cell death induced by arginase and asparaginase differs in sensitivity to amino acids, caspase inhibitors, PKC-activator phorbol myristate, and autophagy inhibitor chloroquine. BCL-2 overexpression prevents arginase-induced cell death, but not arginase-induced cytostasis, implying different mechanisms, with implications for resistance to therapy.

## Materials and methods

### Cell culture and reagents

Six hundred ninety-seven cells are a childhood pre-B lymphoblastic cell line [31] and were purchased from the European Cell Culture Collection (who verified cellular identity by short tandem repeat profiling). 697 cells stably infected with control retrovirus (697-Neo), or recombinant Bcl-2 containing retrovirus (697-BCL2) were kindly provided by Professor Miyashita [32]. Ramos and DG-75 cells were kindly supplied by Dr Suzanne Turner (Department of Pathology, University of Cambridge). All cells were passaged for fewer than 6 months after receipt or resuscitation.

Primary cells were isolated from buffy coats (white cell rich blood units) obtained from the UK National Blood Service. They were lymphocyte enriched by Ficoll separation, and monocyte depleted by adhesion. Both were cultured in RPMI 1640 (PAA Laboratories) supplemented with 10% foetal bovine serum (FBS, Life Technologies) and 1% penicillin/streptomycin (PAA) at 37 °C in a humidified atmosphere maintained at 5% CO_2_.

Bovine arginase (NBS Biologicals, UK) was dissolved in phosphate buffered saline (PBS) since activity assays of NBS arginase were similar in PBS and manganese-maleic acid buffers. Recombinant human pegylated arginase was used where indicated (gift from Cheng [33]). Asparaginase, derived from *E. coli*, was also dissolved in PBS.

For arginase-pretreated media, arginase was added to RPMI for 48 h and incubated at 37 °C. Enzyme was removed by centrifuging media through an Amicon Ultra centrifugal filter (10 kDa cut-off, Millipore, sterilised by UV irradiation and 3 sterile PBS washes) and then supplementing with dialysed FBS. Lack of arginase in this medium was confirmed by assaying arginase activity. For asparaginase-pretreated media, asparaginase was added to RPMI for 48 h at 37 °C, and then enzyme removed by centrifugation, and supplementing with dialysed FBS as above.

Unless otherwise stated all chemicals were from Sigma. Inhibitors or activators were applied 30 min prior to any subsequent treatment, and included: *N*-ω-hydroxy-l-norarginine (nor-NOHA, Bachem), L-NAME, *N*-ω-nitro-l-arginine, l-canavanine and L-NMMA (Alexis), Boc-D(OMe)-fmk (BAF, Enzo Life Sciences), Z-VAD-fmk (ZVAD), Z-DEVD-fmk (DEVD) and Z-IETD-fmk (IETD) from Bachem, and salubrinal (Alexis).

### Flow cytometry

Cell death was analysed with an Accuri C6 flow cytometer (BD Biosciences) after staining with Annexin V-FITC conjugate (ImmunoTools) and propidium iodide (PI). Results were analysed using the Accuri software. Unstained and single stained untreated and treated samples for each run were used for gating and compensation.

### Fluorescence microscopy

Cells were stained with Hoechst 33342 and PI, before imaging with a Leica DMI6000 CS microscope (HCX P1 Fluotar 20×/0.40 dry objective). Total cell number, number with condensed nuclei (apoptotic) and number PI positive (necrotic) were assessed.

### Caspase assay

Cellular caspase-3 activity was determined 24 h after treatments by assaying cleavage of DEVD-amc as in Borutaite and Brown [34].

### Western blots

Cells were lysed using buffer (pH 7.4, 10 mM Tris–HCl, 150 mM NaCl, 1 mM EDTA, 0.5% w/v Triton X-100, 0.1% w/v SDS, 0.1% w/v sodium deoxycholate, protease inhibitor cocktail (Roche), phosphatase inhibitors 1 and 2) and supernatants prepared to standard protein concentrations with deionised water and NuPage LDS sample buffer (Life Technologies), incubated and loaded onto 10% Bis-Tris mini gels (Life Techologies). After transferring to nitrocellulose membranes (Life Technologies), blots were blocked with commercial buffer (Odyssey) before incubating with primary antibodies at concentration 1:15,000. After washing and incubating with secondary antibody at concentration 1:10,000, fluorescence was measured with Li-Cor Odyssey CLx.

### Statistical analysis

For all experiments all conditions were repeated at least in duplicates with at least three independent repeats. Statistical analysis was performed with GraphPad Prism version 3.0. Means were compared with one-way ANOVA and post-hoc Bonferroni tests. *p* values < 0.05 were considered significant.

## Results

### Arginase and asparaginase induce death of lymphoblasts via arginine- and asparagine-deprivation

**Fig. 1:**
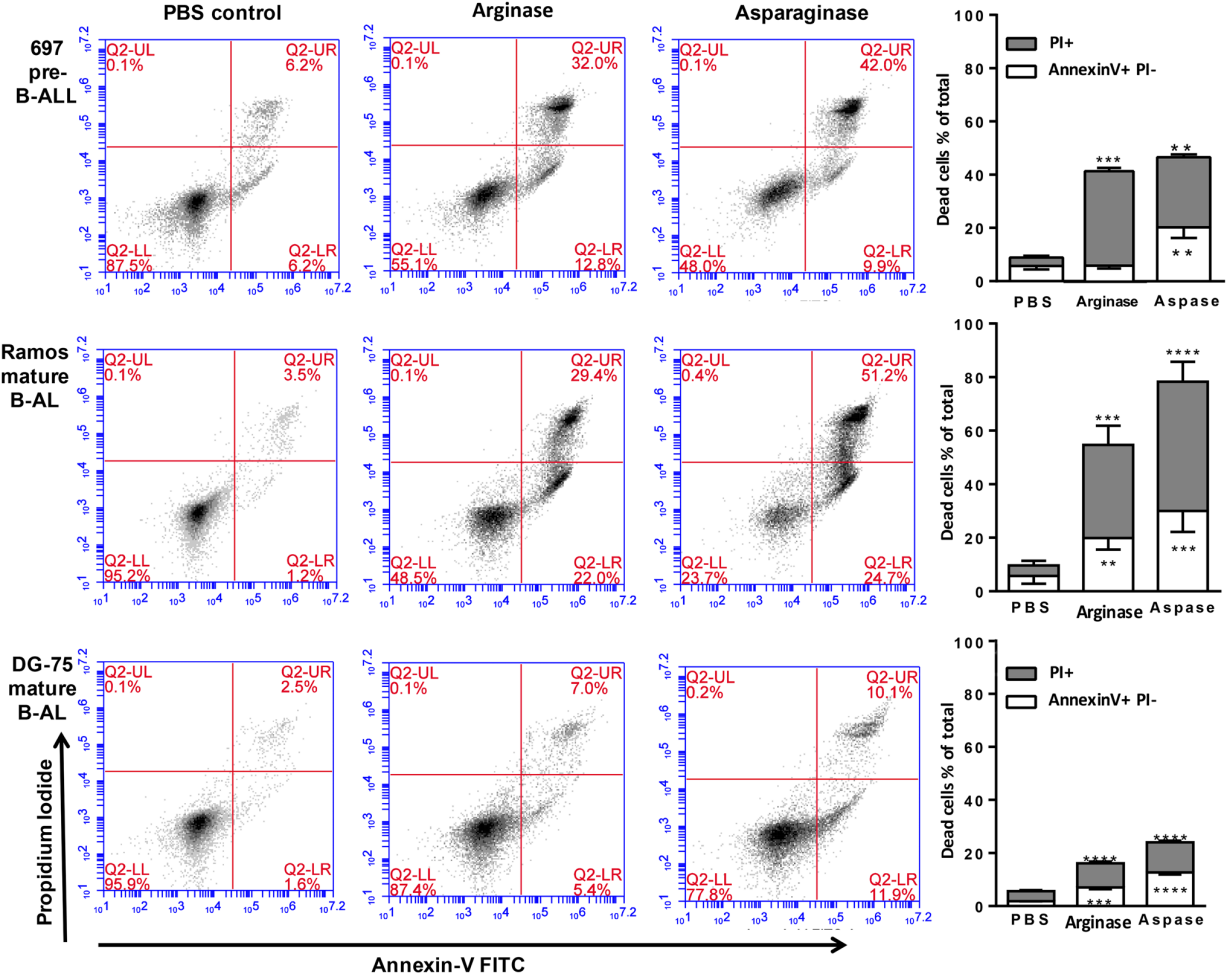
Arginase and asparaginase induce pre-B-ALL and mature B lymphocyte cell death. 697 cells (top row), Ramos cells (middle row), or DG-75 cells (bottom row) were treated for 48 h with either: saline (PBS control, first column), 10 U/mL pegylated human recombinant arginase (second column), or 5 U/mL asparaginase (third column). Death was assessed by flow cytometry after staining with Annexin V-FITC and propidium iodide (PI), counting at least 10,000 events in the gated area. Gating was guided by unstained and single stained treated controls. Representative plots shown for 697, Ramos and DG-75 cells (first three columns), and mean data for at least triplicate repeat experiments (fourth column). Bars are mean ± SEM. **/***/****p < 0.01 / 0.001/0.0001 compared with PBS control. Analysed by one-way ANOVA with post-hoc Bonferroni

The ability of arginase and asparaginase to induce cell death was investigated in three different cell lines derived from B cell malignancies. Cell death could be efficiently induced by arginase or asparaginase in a pre-B-ALL cell line 697 and a mature B lymphoma cell line, Ramos (Fig. 1). Whereas another mature B lymphoma cell line, DG-75 cell, was less sensitive to either enzyme, but cell death could still be significantly induced (Fig. 1).

**Fig. 2:**
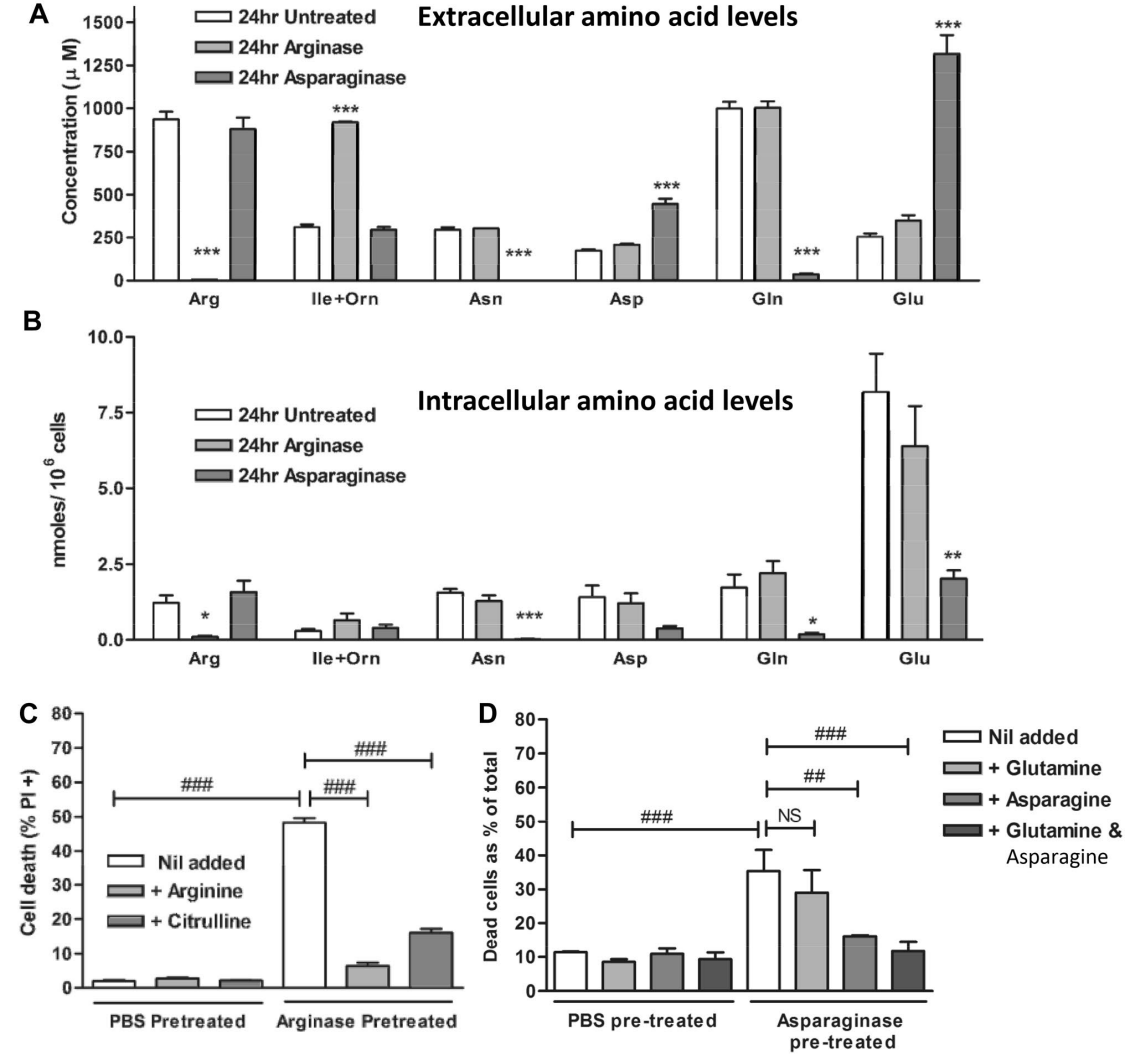
Arginase kills 697 cells by arginine depletion, and asparaginase kills by asparagine, not glutamine, depletion. **a** 697 cells were either untreated or treated for 24 h with 10 U/mL bovine arginase or 5 U/mL asparaginase. Cells were separated from media by centrifugation, washed in PBS and cellular amino acids extracted with cold methanol. Samples were analysed by HPLC. Extracellular **a** and intracellular **b** amino acid levels are shown as means ± SEM for 3 independent experiments performed in duplicate. */**/***p < 0.05/0.01/0.001 compared with the untreated level of the same amino acid. Analysed by two-tailed T-test. *Arg* arginine, *Ile&Orn* isoleucine & ornithine (indistinguishable by this method), *Asn* asparagine, *Asp* aspartate, *Gln* glutamine, *Glu* glutamate. **c** RPMI cell culture medium was pre-treated with 10 U/mL arginase or PBS vehicle control for 48 h, and then the arginase filtered out, serum added ± arginine or citrulline (0.2 g/L as in RPMI). 697 cells were incubated in these media for 72 h, and then viability assayed as before. **d** RPMI was pre-treated for 48 h with 5 U/mL asparaginase or PBS control, and then the asparaginase filtered out, serum added ± asparagine (0.05 g/L), glutamine (0.3 g/L) or both. 697 cells were incubated in these media for 72 h, and then viability assayed as before. Data shown as means ± SEM. N ≥ 3. Analysed by one-way ANOVA with post-hoc Bonferroni. ^##/###^p < 0.01/0.001 for specific comparisons as indicated. *NS* not significant

In order to test whether arginase or asparaginase induced cell death by amino acid depletion, we treated 697 cells with arginase or asparaginase for 24 h (at levels sufficient to induce cell death at 48 h) and measured extracellular and intracellular amino acid levels. Arginase specifically depleted extracellular and intracellular arginine, without affecting other amino acid levels except ornithine, which was elevated, consistent with arginase converting arginine to ornithine (Fig. 2a, b). The finding that arginase almost completely depleted intracellular arginine is consistent with arginase killing these cells by arginine deprivation.

Asparaginase treatment removed extracellular asparagine and glutamine, and elevated extracellular aspartate and glutamate, consistent with asparaginase having both asparaginase activity (converting asparagine to aspartate) and glutaminase activity (converting glutamine to glutamate) (Fig. 2a, b). The intracellular levels of both asparagine and glutamine were severely depleted, consistent with asparaginase inducing death via either asparagine or glutamine deprivation (or both).

In order to test whether arginase was inducing cell death via arginine deprivation, cell culture media were treated with arginase and then the arginase removed (verified by measuring arginase activity), and cells treated with this media ± arginine. Arginase pre-treated media efficiently induced cell death, and the addition of arginine prevented death (Fig. 2c), indicating that arginase induces cell death by arginine deprivation. Citrulline is a precursor for arginine synthesis by cells, so we tested whether citrulline could prevent cell death induced by arginase pre-treated media. Citrulline strongly inhibited cell death induced by arginase pre-treated media, although not as strongly as arginine (Fig. 2c), consistent with arginase inducing cell death by arginine deprivation and citrulline being a precursor for arginine synthesis. This suggests that although arginase might be used to kill B-ALL lymphoblasts, dietary-derived arginine or citrulline may reduce efficacy.

In order to test whether asparaginase was inducing cell death via asparagine or glutamine deprivation, cell culture media were treated with asparaginase and then the asparaginase removed, and cells treated with this media ± asparagine or glutamine or both. Asparaginase pre-treated media efficiently induced cell death, and the addition of asparagine, but not glutamine, prevented death (Fig. 2d), indicating that asparaginase induces cell death by asparagine deprivation, not glutamine deprivation.

### Arginase and asparaginase induce apoptosis, and BCL-2 overexpression prevents cell death but not arginase-induced cytostasis

**Fig. 3:**
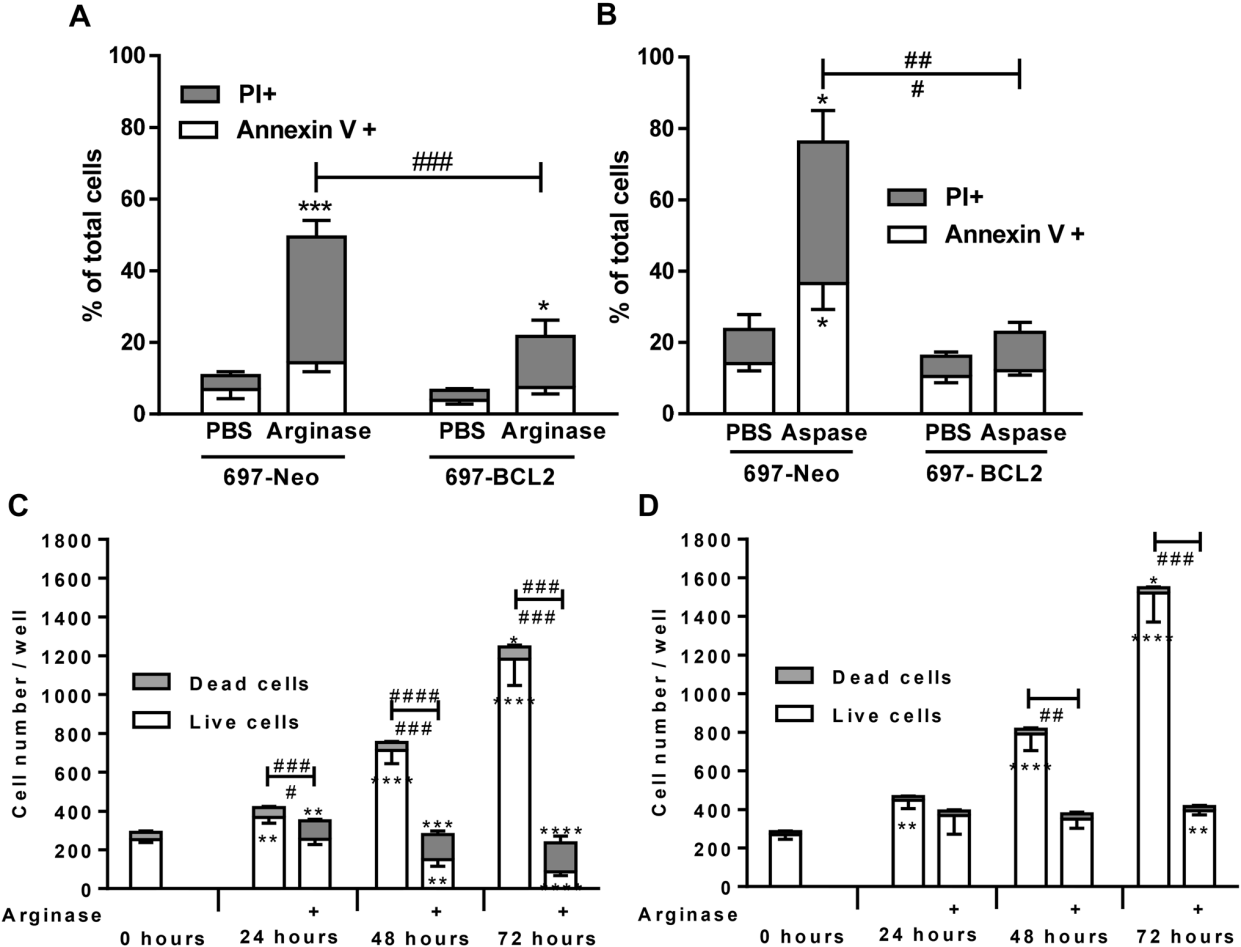
Arginase and asparaginase-induced cell death is blocked by BCL-2 over-expression; but BCL-2 overexpression does not prevent arginine-induced cytostasis. 697-Neo and 697-BCL2 cells were treated with PBS or **a** 10 U/mL arginase or **b** 5 U/mL asparaginase (Aspase) for 48 h. Viability assessed by flow cytometry as before. N ≥ 3. Data shown for apoptotic (Annexin +, PI−) and necrotic (PI+) cells, shown as means + SEM. */***p < 0.05/0.001 compared with PBS control, ^#/##/###^p < 0.05/0.01/< 0.001 specific comparisons as indicated. Analysed by one-way ANOVA with post-hoc Bonferroni. **c** 697-Neo or **d** 697-BCL2 cells were treated with PBS control or 10 U/mL arginase. Viability was assessed by fluorescence microscopy after staining with Höechst and PI. Cells that were PI positive and/or had nuclear condensation were counted as dead, but very few cells had nuclear condensation only. The total cell number for four fields of view for each of 2 wells per condition was counted. Data is shown as means ± SEM for 3 independent experiments. */**/***/****p < 0.05/0.01/0.001/0.0001 for live or dead cells compared with control cells at 0 h or ^#/##/###/####^p < 0.05/0.01/0.001/0.0001 for comparison as shown, dead cells on top, live cells under the bars

After arginase or asparaginase treatment, most cells had condensed nuclei and phosphatidylserine exposure, typical of apoptosis. However, most of these cells were also propidium iodide (PI) positive, indicating plasma membrane rupture, typical of necrosis (Fig. 1). Necrosis can be the primary cause of cell death, or secondary to apoptosis, as apoptosis eventually ruptures the plasma membrane [35]. Apoptosis is mediated by BCL-2 homologous proteins and/or caspases, and apoptotic cell death is blocked by BCL-2 overexpression and/or caspase inhibitors [35]. To distinguish necrotic from apoptotic cell death, 697 cells permanently transfected with *BCL-2* under a constitutive promoter (697-BCL2 cells) were used. Their BCL-2 protein expression is 20-fold higher than control transfected cells 697-Neo [32]. 697-BCL2 cells were relatively resistant to arginase-induced death (Fig. 3a), and completely resistant to asparaginase-induced death (Fig. 3b).

The time course of cell proliferation and cell death ± arginase ± BCL-2 overexpression was followed. Figure 3c shows that in the absence of arginase the cells proliferated with a doubling time of roughly 36 h, and cell death was < 10%. In the presence of arginase, the cells failed to proliferate, and cell death progressively increased from about a quarter at 24 h, to a half at 48 h, and three quarters at 72 h (Fig. 3c). In the cells overexpressing BCL-2, arginase induced no cell death, but proliferation was halted (Fig. 3d). This indicates that arginase induces cell death and cytostasis in lymphoblasts by different mechanisms (one sensitive to BCL-2, and the other not).

### Arginase and asparaginase induce caspase activation, and caspase inhibitors prevent cell death

**Fig. 4:**
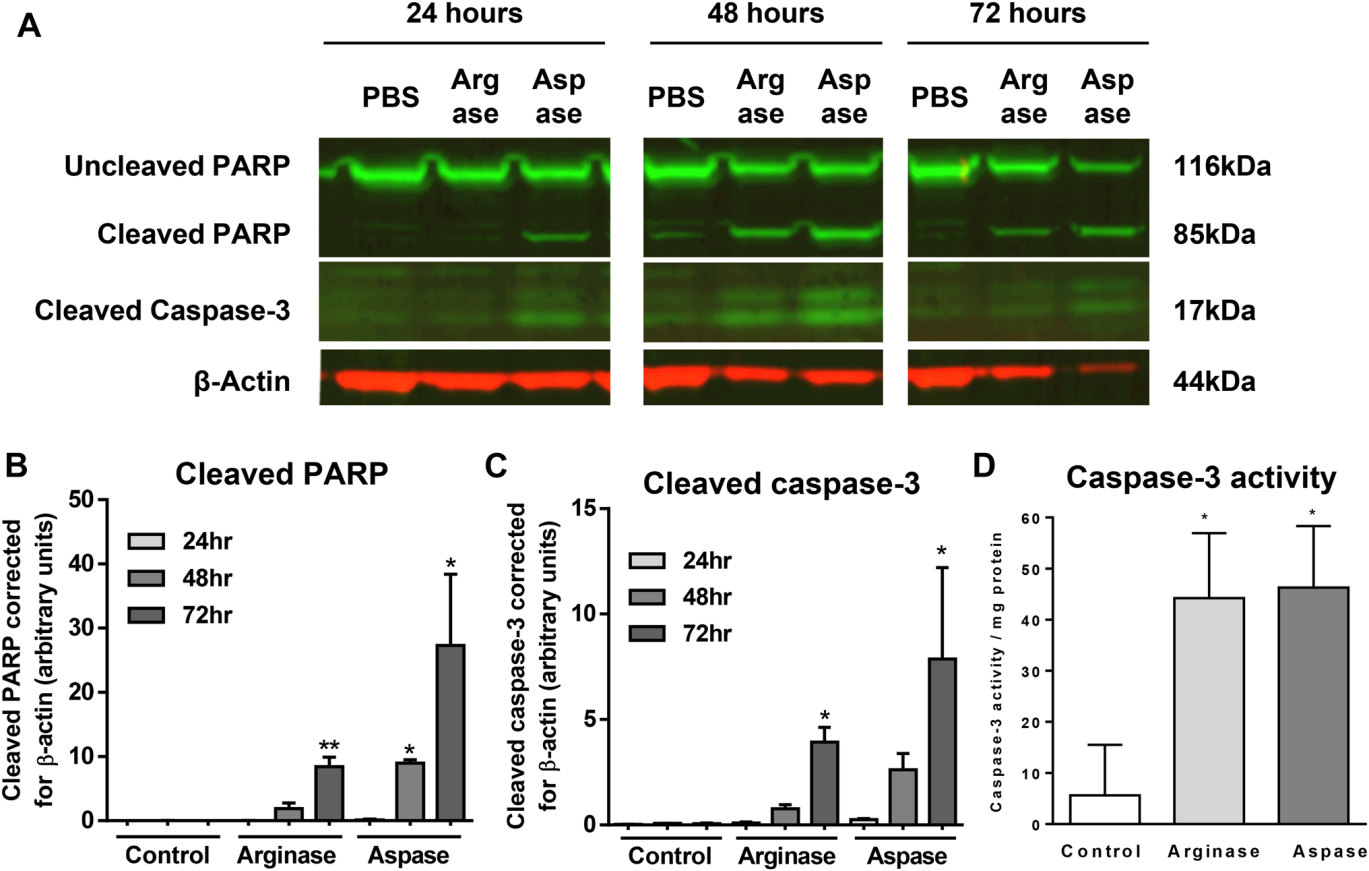
Arginase and asparaginase induce cleavage of PARP, cleavage caspase-3 and caspase-3 activity. **a, b** and **c** 697 cells were treated for the time periods shown with either 10 U/mL arginase, 5 U/mL asparaginase or PBS control. Expression of whole and cleaved PARP, and cleaved caspase-3 was then analysed in lysates. β-actin used as a loading control. N = 2. */**p < 0.05/0.01 compared with PBS control. Analysed by one-way ANOVA with post-hoc Bonferroni. **d** 697 cells were treated with 10 U/mL arginase, 5 U/mL asparaginase or PBS for 24 h, then lysed. Lysates were incubated with ± caspase-3 inhibitor 20 nM DEVD-CHO for 10 min, then caspase-3 substrate Ac-DEVD-amc (11 µM), and the rate of fluorescence increase was followed over 3 h. N ≥ 3. *p < 0.05 compared with PBS control. Analysed by one-way ANOVA with post-hoc Bonferroni

During apoptosis, caspase-3 activity is normally induced by cleavage of inactive pro-caspase 3 to the mature, active form, which is then able cleave endogenous substrate poly(ADP-ribose) polymerase (PARP). So, 697 cell lysates were analysed for PARP and caspase-3 cleavage after treatment with arginase and asparaginase (Fig. 4). This showed substantial increases in cleavage of both PARP and caspase-3 induced by both arginase and asparaginase, supporting the finding that these treatments induce apoptosis.

Cellular caspase-3 activity was measured 24 h after exposure to arginase or asparaginase by measuring the fluorescence released from caspase-3 substrate Ac-DEVD-amc. Both arginase and asparaginase strongly increased the cellular caspase-3 activity (Fig. 4d), consistent with inducing apoptosis.

**Fig. 5:**
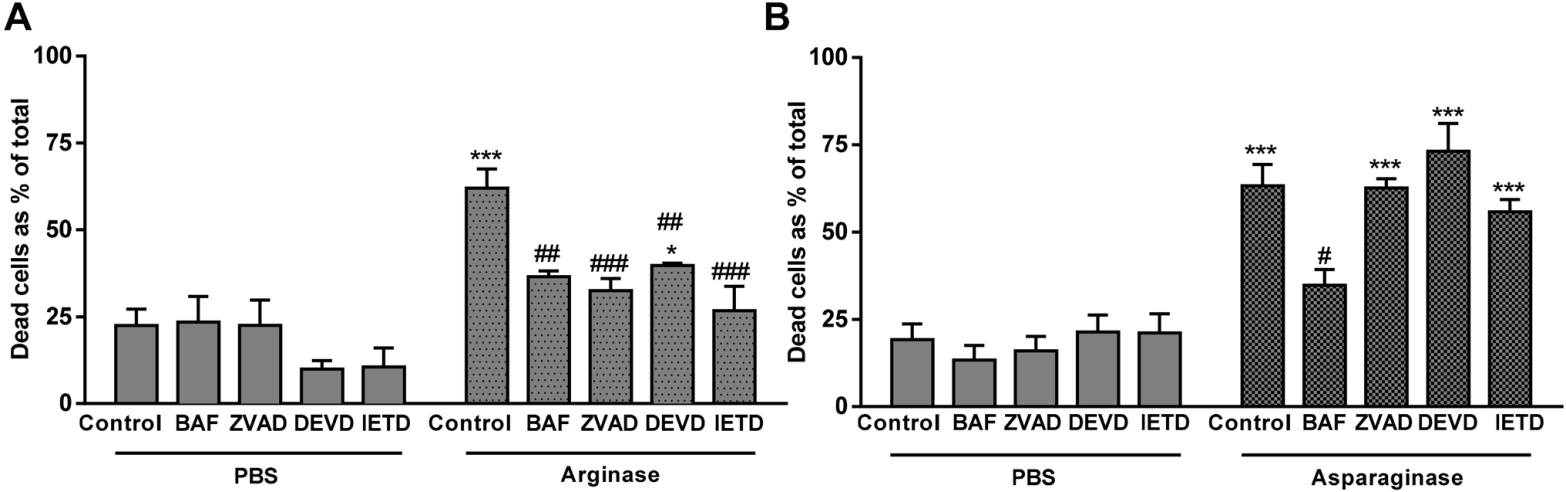
Caspase inhibitors protect against arginase- and asparaginase-induced death. 697 cells were treated with inhibitors 30 min prior to addition of either **a** 10 U/mL arginase or **b** 5 U/mL asparaginase or PBS vehicle control. Caspase inhibitors were: BAF 100 µM, ZVAD 200 µM, DEVD-fmk 200 µM, IETD-fmk 200 µM or DMSO vehicle control. Viability was assessed by flow cytometry at 48 h as before. Data shown as means ± SEM. N ≥ 3. */***p < 0.05/0.001 compared with PBS control, ^#/##/###^p < 0.05/<0.01/< 0.001 compared to either arginase control (**a**) or asparaginase control (**b**). Analysed by one-way ANOVA with post-hoc Bonferroni

Although an induction of caspase-3 activity indicates apoptosis, it does not necessarily indicate that cell death is mediated by apoptosis, because (i) caspase-3 activation can be reversible, and (ii) caspase-3 activation can occur in parallel with other forms of cell death [36]. Thus, in order to test whether death is mediated by caspases, it is important to demonstrate that caspase inhibition prevents death. Various caspase inhibitors of differing specificity were added to cells treated with arginase or asparaginase (Fig. 5). Arginase-induced death was strongly or completely prevented by an inhibitor of caspases and other cysteine proteases (BAF), a pan-caspase inhibitor (z-VAD-FMK), a caspase-3/7 inhibitor (z-DEVD-FMK) or a caspase-8 inhibitor (z-IETD-FMK) (Fig. 5a). Asparaginase-induced death was prevented only by BAF, a relatively non-specific, caspase inhibitor (Fig. 5b). Thus, both arginase- and asparaginase-induced cell death appears to be apoptotic, both with different caspase requirements.

### Arginase-induced cell death is not due to nitric oxide or intracellular polyamine deprivation

**Fig. 6:**
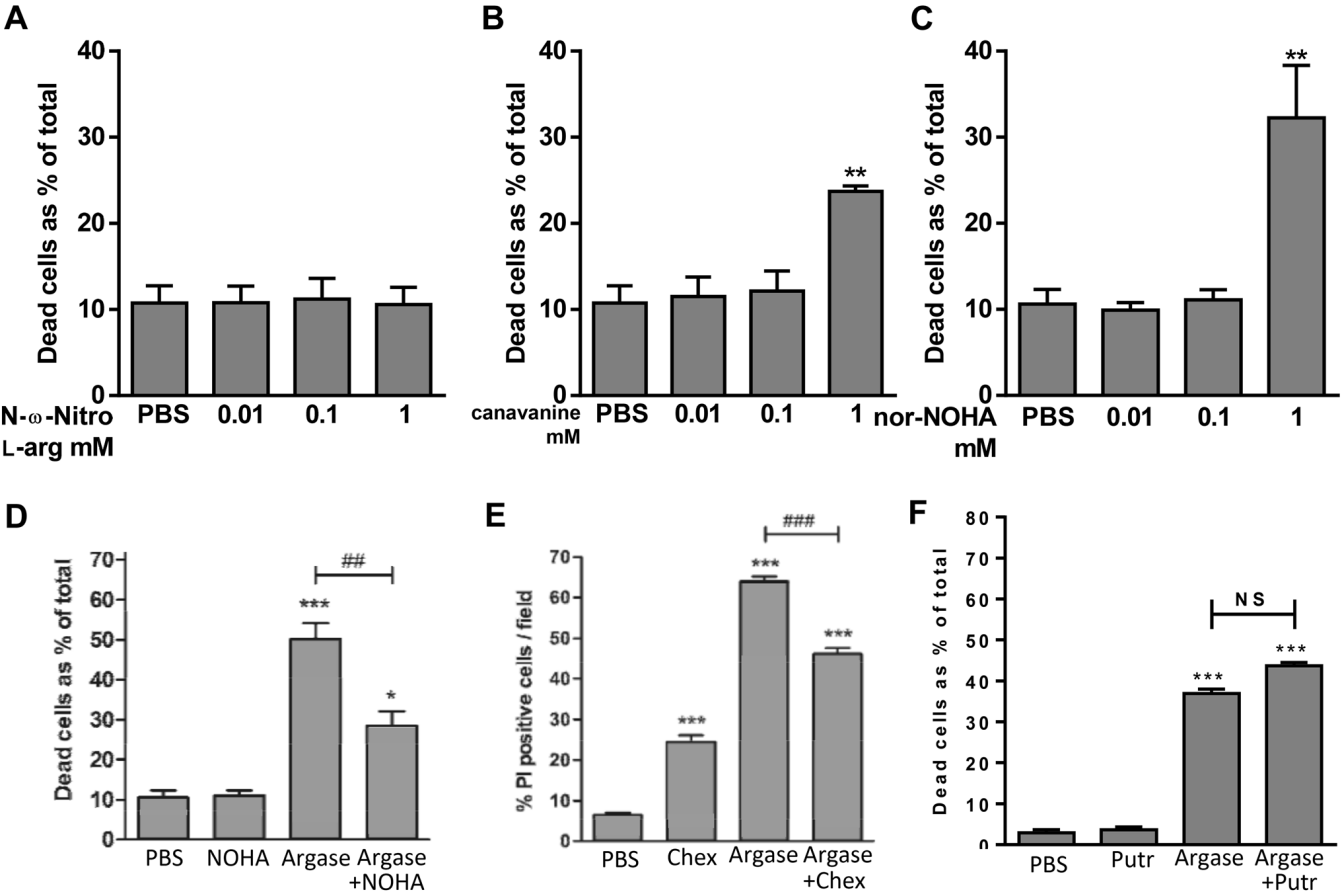
nor-NOHA and l-canavanine can induce cell death, while nor-NOHA and cycloheximide can inhibit arginase-induced death. 697 cells/mL were treated for 48 h with increasing doses of **a***N*-ω-nitro-l-arginine, **b**l-canavanine, or **c** nor-NOHA. 697 cells were treated for 48 h with either 10 U/mL arginase (Argase) or PBS vehicle control ± **d** 0.1 mM nor-NOHA (NOHA), **e** 10 µM cycloheximide (Chex), or **f** 100 µM putrescine (Putr). Viability was assessed by flow cytometry with Annexin V and PI after 48 h. Data is shown for dead cells (Ann+, PI− and PI+) as means + SEM for at least 3 independent experiments performed in triplicate. Analysed by one-way ANOVA with post-hoc Bonferroni. */**/***p < 0.05/0.01/0.001 compared with PBS control, ^##/###^p < 0.01/0.001 as indicated. NS = p > 0.05

L-arginine is a substrate of NO synthesis, and arginine deprivation might therefore induce cell death by inhibition of NO synthesis. We examined this using five arginine analogues with different modes of action (Fig. 6). *N*-ω-nitro-l-arginine, L-NAME and L-NMMA are NOS inhibitors, which had no effect of cell death at 0.01, 0.1 and 1.0 mM (only *N*-ω-nitro-l-arginine shown, Fig. 6a). l-Canavanine is incorporated into proteins in place of arginine, and induced some cell death only at 1 mM (Fig. 6b). nor-NOHA is an arginase inhibitor, and induced cell death only at high dose (Fig. 6c). Thus, as inhibition of NOS does not induce cell death, it seems unlikely arginase induces cell death by inhibiting NOS.

In order to verify that arginase-induced cell death required arginase activity, we treated 697 cells with arginase ± an arginase inhibitor (0.1 mM nor-NOHA) for 48 h. Cell death induced by arginase was inhibited by the arginase inhibitor (Fig. 6d), indicating that the cell death induced by arginase was due to arginase activity, rather than the protein or some contaminant.

Arginase may inhibit protein synthesis as a result of arginine deprivation, but arginase-induced apoptosis might require protein synthesis, so we tested whether inhibiting protein synthesis directly with cyclohexamide would induce cell death, or inhibit that induced by arginase. Cyclohexamide alone did induce some cell death (though less than arginase), and did partially inhibit arginase-induced death (Fig. 6e). This is consistent with arginase inducing cell death partly via inhibition of protein synthesis, but that execution of that death partly depends on protein synthesis.

Intracellular arginine breakdown by arginase releases ornithine, the substrate for polyamine (putrescine, spermidine and spermine) synthesis, compounds vital for cell survival and proliferation [37]. Breakdown of extracellular arginine by extracellular arginase may deprive the cell of polyamines, inducing cell death. To test for a role of this pathway, 697 cells were co-treated with arginase and putrescine, which can replete cell polyamines, but there was no protection against arginase-induced death (Fig. 6f), suggesting polyamine depletion is not responsible for arginase-induced death.

### An autophagy inhibitor, chloroquine, had no effect on arginase-induced death, but increased asparaginase-induced death

**Fig. 7:**
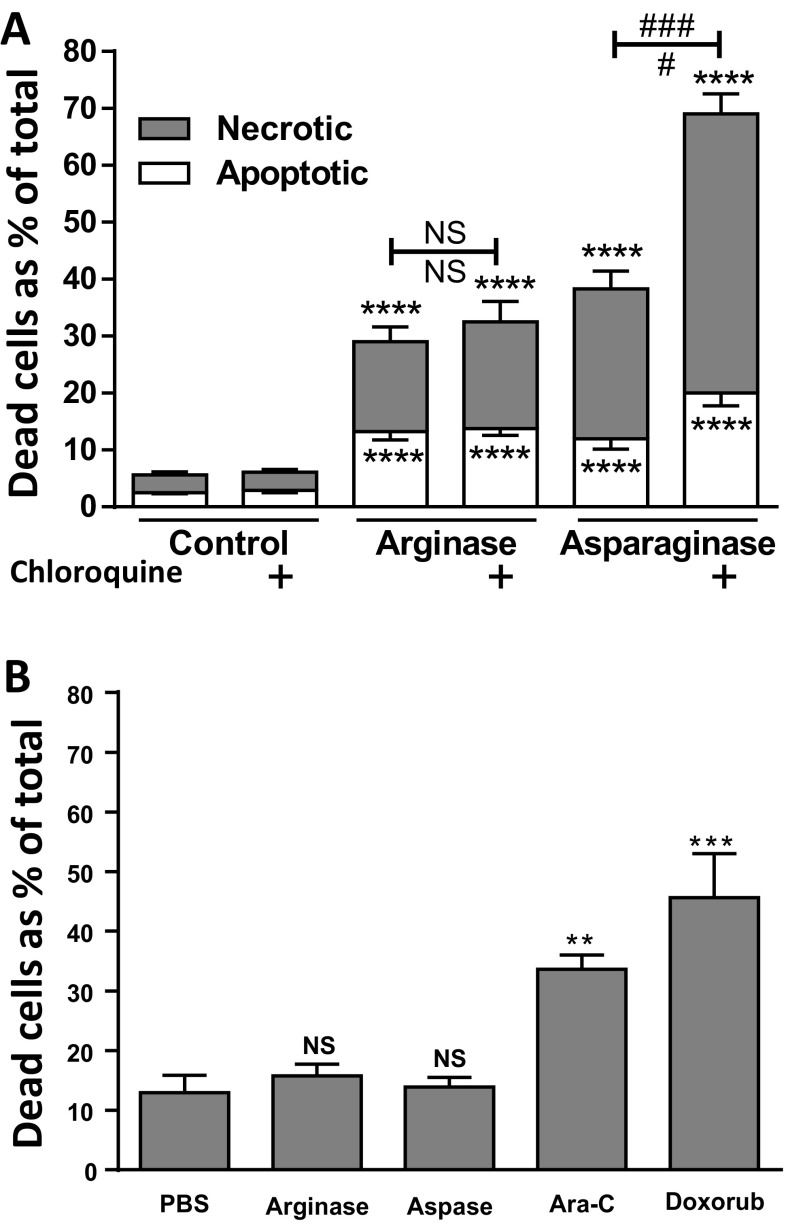
Autophagy inhibitor, chloroquine, augments asparaginase- but not arginase-induced cell death. Neither arginase nor asparaginase are toxic to normal human lymphocytes. **a** 697 cells were treated with 10 U/mL arginase, 5 U/mL asparaginase or PBS control ± 10 µM chloroquine. Death was assessed after 48 h as apoptotic (annexin V+, PI−) or necrotic (PI+). Data is shown as means ± SEM for 3 independent experiments performed in triplicate. ****/**/*p < 0.0001/0.01/0.05 compared with control, #/##/###p < 0.05/0.01/0.001 for specific comparisons as indicated, NS: p > 0.05. Analysed by unpaired two tailed T-test. **b** Normal lymphocytes were enriched from buffy coat of pooled, human blood donors, and plated at 10^6^ cells/mL. 24 h later the following treatments were added: PBS, 10 U/mL bovine arginase, 5 U/mL asparaginase (Aspase), 10 µg/mL cytarabine (Ara-C) or 2 µM doxorubicin (Doxrub). The proportion of dead cells was assessed 48 h later by flow cytometry as the % of cells that were either propidium iodide + (necrotic) or Annexin V+, propidium iodide (apoptotic). Bars are means ± SEM for at least 3 independent experiments each performed in triplicate. Analysed by one-way ANOVA with post-hoc Bonferroni. **/***p < 0.01/0.001 compared with PBS control. *NS* not significant

Autophagy can be induced by amino acid deprivation, and may stimulate or protect against cell death. We tested whether a clinically-available autophagy inhibitor, chloroquine, affected the cell death induced by arginase or asparaginase. Chloroquine treatment alone had no effect on cell death of 697 cells, and had no effect on the cell death induced by arginase, however, chloroquine almost doubled the cell death induced by asparaginase (Fig. 7a). Thus, autophagy does not appear to mediate cell death induced by either enzyme, rather autophagy may protect against asparaginase-induced death, but not arginase-induced death.

### Arginase and asparaginase do not induce cell death of normal blood lymphocytes

In order to test whether normal blood lymphocytes were sensitive to arginase or asparaginase, mixed blood lymphocytes were isolated from healthy humans, and treated with arginase or asparaginase (at concentrations that induced death of lymphoblasts). Neither enzyme induced cell death of lymphocytes, whereas two drugs known to kill lymphocytes, cytarabine (Ara-C) or doxycycline, did induce cell death (Fig. 7b). These data indicate that arginase or asparaginase specifically kill a subset of malignant B cells, without killing primary lymphocytes, and thus are potentially therapeutic.

## Discussion

Interest in arginine depletion as a potential treatment for several solid tumours has accelerated, but there have been relatively few studies in haematological malignancies and very few in precursor-B ALL. We have shown here that arginine depletion induces apoptotic death in the childhood pre-B ALL cell line 697. We have also shown that arginase induces cell death through depletion of arginine alone and that death is inhibited by arginine or citrulline replacement, or an arginase inhibitor. As cell death is reduced by citrulline supplementation, 697 cells probably express arginosuccinate synthetase, and thus may be resistant to arginine deiminase.

Both arginase and asparaginase efficiently induced cell death in a mature B cell line, Ramos, derived from a patient with Burkitt lymphoma, whereas both enzymes were relatively inefficient in inducing cell death in another such cell line, DG-75. The latter cell line is known to be relatively resistant to apoptosis due to a frameshift mutation in BAX [38]. As the intrinsic pathway of apoptosis is dependent on BAX (or BAK), this mutation may contribute to the relative resistance of DG-75 cells to arginase and asparaginase treatment. As Burkitt lymphoma lymphoblasts do not normally overexpress BCL-2 or other mutations rendering them resistant to apoptosis, arginase and asparaginase may be useful in the treatment of Burkitt lymphoma.

Asparaginase has been used for many years in the treatment of ALL without its mechanism of action being fully understood [30]. It has been shown that asparaginase also has glutaminase activity, which can kill cancer cells [39–42], raising the question of how important glutamine depletion is to the induction of leukaemic cell death. We found that asparaginase did indeed deplete both asparagine and glutamine from media and cells. However, the death results from asparagine depletion, without any contribution from glutamine depletion, since cells could be rescued by asparagine but not glutamine replacement.

Our results indicate an apoptotic mode of death for arginase-treated 697 cells as caspase-3 activity was increased and cell death prevented by caspase inhibitors or BCL-2 overexpression. Most arginase-treated cells were both Annexin V positive (indicating phosphatidylserine exposure) and propidium iodide positive (indicating permeable plasma membrane) and had condensed nuclei. These cells may progress rapidly from caspase activation and nuclear condensation to loss of plasma membrane integrity, reflecting rapid secondary necrosis. Our finding that protein synthesis inhibition partially inhibits arginase-induced cell death is also consistent with an apoptotic mode of death via induced expression of death-inducing gene. We found that a caspase-8 inhibitor (IETD), as well as caspase-3 and non-specific caspase inhibitors, completely prevented arginase-induced death. This suggests the involvement of death receptors or other receptors linked to caspase-8. Others [43] have demonstrated the importance of caspase-8 to cytotoxicity of various chemotherapeutic agents in various leukaemic cell lines.

The cell death induced by asparaginase has been described as apoptotic on the basis of G1 arrest, DNA fragmentation, morphological changes [44, 45], phosphatidylserine externalisation, caspase-3 activation, PARP cleavage [46] and cytochrome c release [47]. Our results agree that asparaginase-induced cell death is apoptotic, based on chromatin condensation, phosphatidylserine externalisation, caspase activation and protection by BCL-2 overexpression. However, although cell death was partially prevented by a cysteine protease inhibitor BAF, it was not prevented by caspase inhibitors ZVAD, DEVD and IETD. BAF can inhibit caspases but also other cysteine proteases, such as cathepsins that can mediate cell death [48], thus it is possible that the protection by BAF is not mediated by caspase inhibition. This may be consistent with asparaginase-induced apoptosis being mediated by apoptosis-inducing factor (AIF), as has been reported for acute myeloid leukemia cells [49]. Whatever the mechanisms, because there are significant differences between the death induced in lymphoblasts by arginase and asparaginase, arginase has the potential to be used as an alternative therapy for asparaginase-resistant leukaemia.

Although BCL-2 overexpressing cells (697-BCL2) are relatively resistant to arginase, their proliferation is completely arrested over prolonged treatment periods. A similar result has been reported with dexamethasone [32], which we have replicated (data not shown). This indicates that arginase induces cell death and cytostasis by two different mechanisms in 697 cells: cell death is blocked by BCL-2 overexpression and therefore is probably mediated by BH3-only domain proteins, whereas cytostasis is not affected by BCL-2 overexpression and therefore is independent of apoptosis. Arginase-induced cytostasis may result from arginine deprivation and consequent lack of protein synthesis for cell proliferation. This agrees with the finding that arginine deprivation-induced death in colorectal carcinoma cells could be blocked by zVAD, but that this did not restore proliferation [50]. Clinically, prolonged plasma arginine deprivation may be necessary to clear leukaemia cells overexpressing BCL-2.

Autophagy can be induced by amino acid deprivation, and may stimulate or protect against cell death. We did not measure autophagic flux as this would not tell whether autophagy stimulated or protected against cell death. However, we found that an autophagy inhibitor, chloroquine, alone had no effect on cell death of 697 cells, but almost doubled the cell death induced by asparaginase. Thus, autophagy appears to protect against asparaginase-induced death. Asparaginase therapy for acute leukaemia could potentially be enhanced by combining with an autophagy inhibitor, such as chloroquine. However, if so, it would be important to test whether such co-treatment was not also toxic to non-cancerous cells.

Importantly, normal lymphocytes were not sensitive to either arginase or asparaginase, even though cell death was induced by cytarabine and doxorubicin treatment. Our results demonstrate that arginase is cytotoxic to malignant B-cells without killing normal lymphocytes.

In summary, arginine depletion by arginase represents a promising treatment approach for leukaemia. It’s mechanism of action differs in important ways from that of asparaginase suggesting that it may be a useful agent in asparaginase-resistant disease or where asparaginase is contra-indicated (e.g. following serious allergic reaction). Arginase induces rapid and potent cell death of B-ALL leukaemic cells by apoptosis, without killing normal human lymphocytes. Arginase kills cells by arginine depletion, while asparaginase kills cells by asparagine depletion, which has implications for future engineering of these enzymes for efficacy, as well as for the dietary regimen of patients undergoing therapy. The ability of asparaginase to kill lymphoblasts may be enhanced by co-treatment with chloroquine. Arginase and asparaginase may be therapeutic in Burkitt lymphoma, but it would be important to test this in primary lymphoma cells from patients.
